# Socioeconomic differences in self-rated oral health and dental care utilisation after the dental care reform in 2008 in Sweden

**DOI:** 10.1186/1472-6831-14-134

**Published:** 2014-11-18

**Authors:** Anu Molarius, Sevek Engström, Håkan Flink, Bo Simonsson, Åke Tegelberg

**Affiliations:** Competence Centre for Health, Västmanland County Council, Västerås, Sweden; Department of Public Health Sciences, Karlstad University, Karlstad, Sweden; Department of Public Health and Caring Sciences, Family Medicine and Preventive Medicine, Uppsala University, Uppsala, Sweden; Public Dental Service, Västmanland County Council, Västerås, Sweden; Centre for Clinical Research, Uppsala University, Västerås, Sweden; Faculty of Odontology, Malmö University, Malmö, Sweden

**Keywords:** Adult, Self-rated oral health, Health inequality, Dental care attendance, Epidemiology, Sweden

## Abstract

**Background:**

The aims of this study were to determine self-rated oral health and dental attendance habits among Swedish adults, with special reference to the role of social inequalities, after the Swedish dental care reform in 2008.

**Methods:**

The study is based on a survey questionnaire, sent to 12,235 residents of a Swedish county, in 2012. The age group was 16–84 years: 5,999 (49%) responded. Using chi-square statistics, differences in prevalence of self-rated oral health and regular dental attendance were analysed with respect to gender, age, educational level, family status, employment status and country of birth. Self-rated poor oral health was analysed by multivarite logistic regression adjusting for the different socio-demographic factors, financial security and having refrained from dental treatment for financial reasons.

**Results:**

Three out of four respondents (75%) reported fairly good or very good oral health. Almost 90% claimed to be regular dental attenders. Those who were financially secure reported better oral health. The differences in oral health between those with a cash margin and those without were large whereas the differences between age groups were rather small. About 8% reported that they had refrained from dental treatment for financial reasons during the last three months. Self-rated poor oral health was most common among the unemployed, those on disability pension or on long-term sick leave, those born outside the Nordic countries and those with no cash margin (odds ratios ranging from 2.4 to 4.4). The most important factor contributing to these differences was having refrained from dental treatment for financial reasons.

**Conclusion:**

The results are relevant to strategies intended to reduce social inequalities in oral health, affirming the importance of the provision of equitable access to dental care.

## Background

By the late twentieth century, the oral health of the Swedish population, especially among children and adolescents, had improved dramatically [1]. As this population ages, a greater proportion of older adults will retain their natural dentitions; thus over time the dental treatment needs of the elderly are expected to increase [2]. Treatment demand and expectations will also change character, influenced by changes in population structure, oral health and development in dental services.

Swedish studies of oral health and dental attendance report that over 80 percent of adults visit a dentist within a two-year period [3]. Regular dental attendance among adults has been shown to be associated with better oral health [4]. A survey of dental attendance patterns of older adults in thirteen European countries indicates that the patterns established during childhood tend to persist throughout life [5]. Socio-economic factors and country of birth are important determinants of both dental attendance habits and failure to seek dental care despite treatment need [6]. In the Swedish context, financial limitations are cited as the most common reason [1] whereas in the UK, cost and anxiety are reported as the most important barriers to dental care [7].

Several studies have disclosed socio-economic differences in self-rated oral health, not only in Sweden [6, 8–10], but also in many other countries [10–14]. Despite the introduction of a dental welfare system as long ago as 1974, social gradients in oral health outcomes persist in Sweden [1]. Contrary to expectations, oral health inequalities are no less in the Scandinavian countries than in other European welfare state regimes [10]. In 2008, a dental care reform was implemented in Sweden. There were two overall objectives [15]: firstly to maintain good oral health for those with little or no dental treatment needs. Especially important was continuing regular dental attendance among 20–29 year-olds, i.e. after they were no longer eligible for free dental care provided for children and adolescents up to school leaving age. The second overall objective was to provide dental treatment for those with extensive needs at reasonable, subsided cost.

Using data from the National Public Health Survey of 2004–2005 in Sweden, Wamala et al. found that 60% of the socioeconomic differences in poor oral health were explained by lack of access to dental care [8]. In their study a combined index was used to measure socioeconomic disadvantage. Donaldson et al. [4], using data from the 1998 Adult Dental Health Survey in the UK, showed in an analysis using structured equation models that low socioeconomic status leads to lower number of sound teeth through barriers to dental attendance and dental attendance profile.

The primary aim of our study was to explore social inequalities in self-rated oral health among adults aged 16–84 years after the Swedish dental care reform in 2008. To our knowledge, this is the first study to investigate these inequalities in Sweden after the reform. In contrast to Wamala et al. who used a combined measure of socioeconomic status, we wanted to know which groups in the general population, in relation to gender, age, educational level, family status, employment status and country of birth, are especially affected. A secondary aim was to analyse, by gender and age group, self-rated oral health and dental attendance habits, with special reference to refraining from treatment for financial reasons. Our hypothesis was that low socioeconomic status leads to poor oral health through financial insecurity and refraining from dental care due to financial reasons.

## Methods

This study is based on data from the population survey “Health on equal terms” conducted in collaboration with the Swedish Institute of Public Health. In the County of Västmanland, this questionnaire was sent to 12,235 people aged 16–84 years: 5,999 (49 percent) responded. The survey was conducted during April-June 2012. The sampling was random and stratified by gender, age group and municipality. The sampling frame was the population register at Statistics Sweden, the statistical administrative authority in Sweden, covering all inhabitants of the county. Data collection was discontinued after two postal reminders failed to elicit a response.

The survey included questions about lifestyle, living conditions, general health and contact with health services. Several questions concerned oral health and dental care. Self-rated oral health was assessed by the question: “How is your oral health?” with response alternatives: very good, fairly good, neither good nor poor, quite poor and very poor. In the analysis, the first two responses were combined as “good oral health”, and the last two as “poor oral health”. Dental attendance was assessed from responses to the question “When did you last visit a dentist/dental hygienist?” The response options were from “ less than a year ago” to “have never been to a dentist/dental hygienist”. There was also an alternative “do not know/can’t remember”. Those who had visited the dentist/dental hygienist less than three years ago were defined as regular dental attenders. 5,961 persons answered the questions on oral health.

The question about refraining from dental visits was: “Have you during the past three months, considered yourself in need of dental care, but refrained from seeking care? ”. A positive response to this question led to a follow-on question: “What was the reason/ reasons for not seeking dental care?”. The response options were “symptoms subsided,” “ financial reasons ”, “dental fear”, “lack of time” and “other reasons” (multiple choices were possible).

Financial security was assessed by a question about cash margin i.e. whether the respondent could access an amount of 15,000 SEK at a week’s notice, to cover unexpected expenses (yes/no). Employment status and family status were based on questionnaire data.

The individuals in the sample were informed that completed questionnaires would be linked to the Swedish official registries through personal identification numbers, in order to access registry information on gender, age, geographic area, educational level and country of birth. The respondents thus gave informed consent to the linking of registry data. Immediately after record linkage, the personal identification numbers were deleted. Statistics Sweden carried out the sampling and data collection and linkage with registry data, and delivered the de-identified data to the county councils. The survey was approved by the Board of Ethics, Uppsala University (EPN 2012/256).

### Statistical analysis

Chi-squared statistics were applied to analyse differences in the prevalence of self-rated poor oral health and failure to seek dental care, with reference to gender, age, educational level, family status, employment status and country of birth. P-values < 0.05 were considered statistically significant. A multivariate logistic regression analysis was also carried out. The results are reported as odds ratios (OR) and 95 percent confidence intervals (95% CI) for self-rated poor oral health, adjusting for gender and age in all models. In the second model, the odds ratios were adjusted for educational level, family status, country of birth, employment status and cash margin. In the final model the results were further adjusted for having refrained from dental treatment for financial reasons during the last three months. In order to exclude the possibility that the socioeconomic differences are explained by regular dental attendance or other reasons for refraining from dental care, these factors were also controlled for in a post-hoc analysis. The analyses were performed using SPSS statistical software, version 20.

## Results

Three out of four respondents (75%) reported good oral health (Table 1). This was more common among women than men (p < 0.001). Younger adults reported slightly better oral health than older adults. Almost one in ten reported fairly poor or very poor oral health and 16% neither good nor poor oral health.

**Table 1:** **Number of respondents, crude prevalence of self-rated good dental health and proportion visiting their dentist/dental hygienist regularly (last visit less than three years ago), stratified according to gender and age group**

		16-34 yrs	35-49 yrs	50-64 yrs	65-84 yrs	Total	p-value ^*^
						16-84 yrs	
N	Women	460	430	663	1134	2687	-
	Men	667	654	804	1149	3274	-
	Total	1127	1084	1467	2283	5961	-
Good oral health (%)	Women	81	76	79	75	78	0.013
	Men	74	75	71	71	72	0.301
	Total	78	76	75	73	75	0.018
Regular dental attender (%)	Women	89	88	93	91	90	0.013
	Men	86	84	88	90	88	0.007
	Total	88	86	90	90	89	0.001

*p-value from chi-square test for difference between age groups.

In total, 89% reported that they were regular dental attenders (Table 1), while 7% of the men and 4% of the women reported rare attendance. There were small but statistically significant differences between age groups and between men and women. Overall, those aged 65–84 years were the most regular dental attenders.

**Figure 1:**
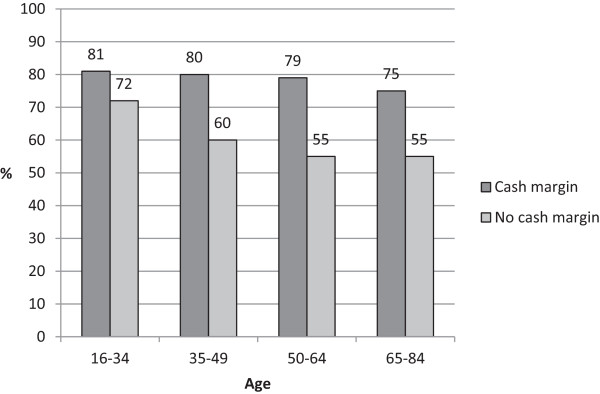
**Prevalence (%) of self-rated good dental health in different age groups among those with and without a cash margin (able to acquire 15,000 SEK in a week).**

Persons with financial security reported the best oral health. The difference between those with an accessible cash margin and those without was greater than differences between age groups. However, both differences were statistically significant (p < 0.05). Among those with a cash margin, the proportion with self-rated good oral health decreased only marginally up to retirement age. Among those lacking a cash margin, the proportion with perceived good oral health was lower and continued to decrease up to the age of retirement (Figure 1).

A total of 9% of the respondents perceived their oral health to be poor (Table 2). A similar proportion had refrained from dental treatment for financial reasons during the last three months. Self-rated oral health and dental care differed in relation to education level, employment status and country of birth. Self-rated poor oral health was most common among the unemployed, those on disability pension or long-term sick leave, those born outside the Nordic countries and those with no cash margin. Refraining from dental treatment for financial reasons was most common in the same groups and among single mothers (Table 2). The proportion who reported other than financial reasons for refraining from dental care was lower. In total, 2% reported that symptoms subsided, 3% reported dental fear, 2% lack of time and 3% other reasons.

**Figure 2:**
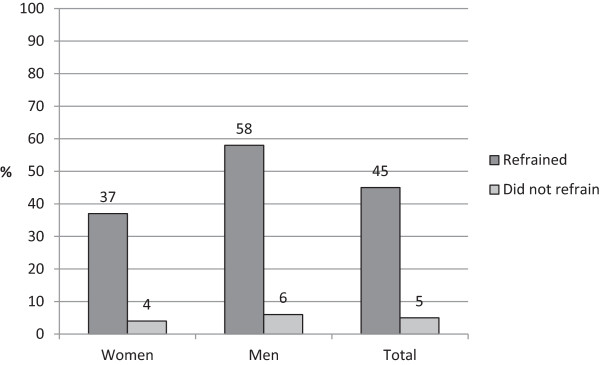
**Prevalence (%) of self-rated poor dental health among those who, during the last three months, refrained from dental treatment for financial reasons and those who did not.**

Self-rated poor oral health and refraining from treatment were also strongly associated: among those who had refrained from treatment for financial reasons during the last three months, the proportion with poor oral health was 45%, compared with only 5% among the remainder of the sample (p < 0.001). Perceived poor oral health was thus around nine times more common among those who had refrained from dental treatment than among those who had not (Figure 2).

**Table 2:** **Socioeconomic disparities in self-rated oral health and dental care**

		***N (%)***	***Poor oral health (%)***			***Refrained from dental treatment for financial reasons during the last three months (%)***		
**Educational level**			*Women*	*Men*	*Total*	*Women*	*Men*	*Total*
	Higher education	1730 (29)	5	7	6	8	5	7
	Upper secondary school	2746 (47)	9	10	9	11	7	9
	Elementary school	1422 (24)	9	12	11	7	8	7
**Family status**								
	Cohabitant without children	3071 (52)	6	7	7	6	4	5
	Cohabitant with children	964 (16)	7	10	8	12	9	11
	Single	1198 (20)	11	17	14	12	13	12
	Single with children	192 (3)	12	15	13	21	9	18
	Other	485 (8)	5	10	7	7	4	6
**Country of birth**								
	Sweden	5058 (85)	7	9	8	8	5	7
	Other Nordic country	461 (8)	11	12	12	13	9	11
	Outside Nordic countries	442 (9)	12	20	16	22	17	20
**Employment status**								
	Employed	2530 (45)	6	8	7	9	6	8
	Self-employed	278 (5)	5	6	6	7	4	5
	Student	433 (8)	5	8	6	14	4	11
	Retired	1880 (33)	8	10	9	4	5	5
	Unemployed	200 (4)	15	21	17	24	21	23
	Disability pensioner	167 (3)	20	21	20	21	23	22
	Long-term sick leave	150 (3)	14	17	15	14	23	16
**Cash margin**								
	Yes	4819 (82)	5	7	6	4	3	4
	No	1089 (19)	17	23	19	27	25	26
**Total**			8	10	9	9	7	8

A multivariate logistic regression analysis of these factors confirmed that the strongest risk factor for self-rated poor oral health was having refrained from dental treatment for financial reasons during the last three months (Table 3). The unadjusted odds for poor oral health were twice as high among people born outside the Nordic countries than among Swedish-born. Similarly, the odds for poor oral health were 2–3 times higher among the unemployed and those on disability pensions or on long-term sick leave than among the employed. After taking into account access to a cash margin and in particular, whether the subject had refrained from dental treatment for financial reasons, these differences attenuated and were no longer statistically significant. Refraining from dental treatment thus largely explained differences in oral health status in relation to country of birth, between the employed and the unemployed or those on disability pension or on long-term sick leave.

**Table 3:** **Odds ratios (95% confidence interval in parenthesis) for self-rated poor oral health**

	OR (95% CI)	OR (95% CI)	OR (95% CI)
	Model 1*	Model 2**	Model 3***
**Educational level**			
Higher education	1 (ref)	1 (ref)	1 (ref)
Upper secondary school	1.8 (1.4, 2.3)	1.5 (1.2,2.0)	1.5 (1.1,2.0)
Elementary school	2.2 (1.7,2.9)	1.5 (1.1,2.0)	1.6 (1.2,2.3)
**Family status**			
Cohabitant without children	1 (ref)	1 (ref)	1 (ref)
Cohabitant with children	1.4 (1.0,1.9)	1.4 (1.0,2.1)	1.5 (1.0,2.2)
Single	2.4 (1.9,3.0)	1.9 (1.4,2.4)	1.7 (1.3,2.2)
Single with children	2.4 (1.5,3.9)	1.6 (0.9,2.7)	1.6 (0.9,2.6)
Other	1.3 (0.9,2.1)	1.1 (0.7,1.8)	1.6 (0.9,2.6)
**Country of birth**			
Sweden	1 (ref)	1 (ref)	1 (ref)
Other Nordic country	1.6 (1.2,2.1)	1.2 (0.9,1.8)	1.0 (0.7,1.5)
Outside Nordic countries	2.3 (1.7,3.0)	1.7 (1.2,2.4)	1.3 (0.9,1.9)
**Employment status**			
Employed	1 (ref)	1 (ref)	1 (ref)
Self-employed	0.8 (0.5,1.4)	0.9 (0.5,1.6)	0.9 (0.5,1.6)
Student	1.0 (0.6,1.6)	0.6 (0.4,1.1)	0.6 (0.4,1.1)
Retired	1.5 (0.9,2.7)	1.3 (0.7,2.4)	1.1 (0.6,2.1)
Unemployed	3.0 (2.0,4.4)	1.4 (0.9,2.2)	1.3 (0.8,2.2)
Disability pensioner	3.8 (2.5,5.7)	1.9 (1.2,3.0)	1.6 (0.9,2.7)
Long-term sick leave	2.6 (1.6,4.3)	1.9 (1.1,3.2)	1.7 (1.0,3.1)
**Cash margin**			
Yes	1 (ref)	1 (ref)	1 (ref)
No	4.4 (3.6,5.3)	3.4 (2.7,4.3)	1.6 (1.2,2.1)
**Refrained from dental care for financial reasons**			
**No**	1 (ref)		1 (ref)
**Yes**	17.6 (13.9,22.2)		12.8 (9.8,16.8)

*Adjusted only for age and gender.

**Adjusted for age, gender and all other variables included in the model, except for having refrained from dental treatment.

***Adjusted for age, gender and all other variables included in the model.

In the last analysis, regular dental attendance and other than financial reasons for refraining from dental care were introduced into the model (data not shown). Of these, regular dental attendance (OR: 3.4; 95% CI: 2.6, 4.5), dental fear (OR: 4.4; 95% CI: 2.9, 6.5) and lack of time (OR: 1.9; 95% CI: 1.0, 3.6) were statistically significantly associated with poor self-rated oral health. The odds ratio for refraining from dental care due to financial reasons attenuated to 7.5 (95% CI: 5.6, 10.0) but it was still the strongest factor for explaining poor oral health.

## Discussion

In total, 75% of the respondents in this study rated their oral health as fairly good or very good. This is consistent with other studies of Swedish adults [1]. Younger adults reported slightly better oral health than older age-groups. In total, 9% rated their oral health as poor. Almost 90% reported that they were regular dental attenders, while 7% of men never or rarely visited the dentist, compared to 4% of women. About 8% had refrained from dental treatment for financial reasons during the last three months. There were, however, large differences among socioeconomic groups with respect to self-rated oral health and dental attendance.

Self-rated poor oral health was most common among the unemployed, those on disability pension or long-term sick leave and those born outside the Nordic countries, as well as people with no cash margin. Having refrained from dental treatment was also most common in the same groups and among single mothers. The higher incidence of perceived poor oral health among disability pensioners and those on long-term sick leave indicates a link between oral and general health. There is now accumulating evidence of the impact of general health on oral health [16–18]. Moreover, among the elderly, medication is more frequent and there is greater co-morbidity of systemic diseases. This may also influence the results [18].

Further analysis showed that differences in oral health in relation to country of birth, employment status, disability pension and long-term sick leave are largely explained by differences in the proportion who refrain from dental treatment for financial reasons. This indicates that among those who had not refrained from dental treatment for financial reasons the prevalence of poor self-rated oral health was about the same irrespective if they were unemployed or employed and irrespective of country of birth. This highlights the importance of finding means of maintaining good oral health among population groups who under the current system refrain from dental treatment for financial reasons. The results also indicate the need for a more detailed analysis to determine how regular dental attendance can be facilitated for risk individuals and risk groups since regular dental attendance is established in childhood [5]. Young adults tend to postpone their dental visits as of low priority. A study of 32 year-olds has disclosed a relationship between low socioeconomic status and poor oral health, tooth loss and sporadic dental treatment [19].

This study is based on the respondents’ self-evaluation of oral health. Earlier studies have shown that that individuals of poor socioeconomic status tend to underestimate their treatment needs [20, 21]. This implies that professionally assessed, objective dental status may disclose even greater socioeconomic differences than those based on self-assessment.

Social inequalities in oral health have been reported from the UK [14, 22], Canada and the US [12], Japan [13], Germany [23], Australia [24] and many other countries [10] with consistently poorer oral health among those with lower income or educational level. In our study, the differences in the prevalence of poor oral health between those with and without a cash margin were large whereas the differences between age groups were rather small. Discordant with a study from the UK where socioeconomic differences were found to be larger at younger ages [22], the difference between those with and without cash margin increased with age up to retiment age in our study. Thus socioeconomic inequalities seem to persist in Sweden, despite the dental care reform of 2008. The dental health care system is still based mainly on fees for service, which contributes to these inequalities. Sweden is not an exception: unequal dental attendance, corresponding to financial status, is reported in all OECD countries [25]. On the other hand, in Norway, inequalities in dental service utilisation have been found only among the elderly [26].

Contrary to what might be expected, oral health inequalities are no less pronounced in the Scandinavian countries than in other European welfare state regimes [10]. One explanation could be the finding of our study that there are large socioeconomic differences in the proportion who refrain from dental treatment for financial reasons. This is important because one of the two aims of the Swedish reform of 2008 was to provide dental treatment for those with extensive needs at reasonable, subsided cost but the reform seems not to have had the desired effect.

The social gradient in both general and oral health highlights the underlying influence of psychosocial, economic, environmental and political determinants [27]. It has been argued that the focus of prevention should be shifted from changing behaviours to addressing the underlying social determinants of population oral health [27]. In oral health as well as in general health, the social gradients are produced by society and therefore avoidable [28]. As shown in our study, inequalities in self-rated oral health were, to a large degree, explained by differences in the proportion who refrain from dental treatment for financial reasons. This is consistent with previous studies where barriers to dental care have been found to contribute to inequalities in oral health [4, 8]. In contrast to the studies of Wamala et al. [8] and Donaldson et al. [4] we could also rule out the possibility that regular dental attendance per sé or other reasons for refraining from dental care would explain socioeconomic inequalities in oral health. In our study, regular dental attendance and refraining from dental care due to dental fear or lack of time did also contribute to socioeconomic differences in poor self-rated oral health, but the most important factor was refraining from dental care for financial reasons. Dental fear was the second most important factor for poor self-rated oral health, which is in line with results from the study in the UK by Hill et al. [7].

The response rate in our study was 49%, which is similar to other population-based studies in Sweden [29]. The response rate was lower among younger than older subjects and in men than in women. The educational level of the respondents was also somewhat higher than the general population of corresponding age. A follow-up study of the non-respondents, conducted in a corresponding study in a neighbouring region, indicated that self-rated poor general health was somewhat more common among non-respondents [30]. Given the association between general and oral health, it can be assumed that poor oral health is more common among non-respondents. Thus in the present study there may be an underestimation of socioeconomic differences in oral health.

The cross-sectional design of the study is a limiting factor as it precludes any inference with respect to causality. Poor oral health may increase the probability of refraining from dental attendance, because more advanced oral disease is likely to be more expensive to treat. The corollary is also true, i.e. refraining from dental treatment may exacerbate poor dental health. In either case this is of concern for oral health status, at both the individual and population levels.

The strength of our study is that it is based on a large general population. It includes both men and women and covers a wide age range, from 16 to 84 years. The study also allowed identification of particularly vulnerable groups, such as the unemployed, those on disability pensions and on long-term sick leave, as well as residents born outside the Nordic countries.

The importance of good oral health is highlighted by recent research disclosing associations between general health and oral health [17, 31]. Increased collaboration between the various stakeholders in the health care system - in which dentistry should be included - would provide better opportunities for prevention. Studies of oral health development and health care consumption patterns in different socioeconomic groups are important, both for monitoring dental health and evaluating strategies intended to reduce inequalities.

## Conclusion

Although most of the study population had self-rated good oral health, major differences were found between socioeconomic groups. These differences have persisted, despite the Swedish dental welfare system and recent dental care reform. The differences in perceived oral health were, to a large degree, explained by differences in the proportion who refrain from dental treatment for financial reasons. The results indicate that in strategies intended to reduce social inequalities in oral health, equitable access to dental care is an important factor. Further studies are needed to determine whether providing a better financial access to dental care in lower socioecomic groups will reduce social inequalities in dental health.
